# A Potential Role for Pro-Inflammatory Cytokines in the Development of Insulin Resistance in Horses

**DOI:** 10.3390/ani2020243

**Published:** 2012-05-02

**Authors:** Jessica K. Suagee, Benjamin A. Corl, Raymond J. Geor

**Affiliations:** 1 Department of Dairy Science, Virginia Tech, Blacksburg, VA 24071, USA; E-Mail: bcorl@vt.edu; 2 Department of Large Animal Clinical Sciences, Michigan State University, East Lansing, MI 48824, USA; E-Mail: geor@cvm.msu.edu

**Keywords:** adipose tissue, horses, inflammation, obesity, pro-inflammatory cytokines, skeletal muscle

## Abstract

Insulin resistance is a metabolic condition involving reduced sensitivity of insulin-sensitive tissues to insulin-induced glucose disposal, including adipose tissue, skeletal muscle, and liver. Insulin resistance occurs in overweight and obese horses, and may increase risk for the development of laminitis. The development of insulin resistance is thought to occur in response to increased production of pro-inflammatory cytokines by adipose tissue in obesity, that then have an inhibitory effect on insulin signaling pathways in multiple tissues. This article reviews current knowledge of the involvement of pro-inflammatory cytokines in the development of insulin resistance in horses and uses data from other species to provide context.

## Abstract (executive summary)

Understanding the mechanisms involved in the development of insulin resistance in horses should enable development of effective treatment and prevention strategies. Current knowledge of these mechanisms is based upon research in obese humans and rodents, in which there is evidence that the increased production of pro-inflammatory cytokines by adipose tissue negatively influences insulin signaling in insulin-responsive tissues. In horses, plasma concentrations of the cytokine, tumor necrosis factor-α, have been positively correlated with body fatness and insulin resistance, leading to the hypothesis that inflammation may reduce insulin sensitivity in horses. However, little evidence has documented a tissue site of production and a direct link between inflammation and induction of insulin resistance has not been established. Several mechanisms are reviewed in this article, including the potential for macrophage infiltration, hyperinsulinemia, hypoxia, and lipopolysaccharide to increase pro-inflammatory cytokine production by adipose tissue of obese horses. Clearly defining the role of cytokines in reduced insulin sensitivity of horses will be a very important step in determining how obesity and insulin resistance are related.

## 1. Introduction

There is information supporting a positive relationship between obesity and the risk of developing laminitis in horses and ponies [1,2,3]. The mechanism explaining this relationship was suggested as early as the 1980’s to be insulin resistance when Jeffcott and Field described reduced tolerance to oral glucose administration in obese, previously-laminitic ponies compared to lean, healthy ponies and horses [1]. Current evidence for a relationship between obesity, laminitis, and insulin resistance has been reviewed elsewhere [4,5]. The purpose of this review is to highlight research describing the hypothesis that increased adipose tissue production of pro-inflammatory cytokines during equine obesity leads to the development of insulin resistance. It is relevant to examine the relationship between inflammation and obesity in horses because in other species cytokines directly impair insulin signaling in tissues that have potential to alter whole body glucose metabolism, such as adipose, skeletal muscle, and liver tissue. These data suggest that increased pro-inflammatory cytokines directly contribute to the development of insulin resistance, and this raises two very important questions in equine science: are cytokines involved in the pathology of insulin resistance, and which tissues produce potentially insulin-resistance-inducing cytokines? 

In human medicine, increased circulating concentrations of inflammatory proteins have demonstrated utility for the prediction of future metabolic disease. For example, elevated concentrations of C-reactive protein (CRP), an acute-phase inflammatory protein, indicate increased risk for obesity-associated disorders [6]. This finding has potential clinical application to horses if researchers can identify an inflammatory protein similar in its ability to predict the risk of metabolic disease. Therefore, our overall goal was to integrate available knowledge about inflammatory proteins in horses into the larger body of mechanistic information that has been described in other species. 

## 2. Production of Pro-Inflammatory Cytokines

Chronic low-grade inflammation is a primary component of obesity-associated-metabolic conditions, such as insulin resistance and diabetes. In humans, low-grade inflammation is caused by increased circulating concentrations of pro-inflammatory cytokines (interleukin [IL]-1β, IL-6, tumor necrosis factor-α [TNF]) and acute phase proteins (serum amyloid A [SAA] and CRP) [7,8,9,10,11,12,13,14]. Because laminitis is an obesity-associated disease in horses, new information from human obesity research led to investigations to determine whether increased systemic inflammation was present in obese horses [1]. In 2007, Vick *et al*. described a positive correlation between obesity and circulating concentrations of TNF in Thoroughbred mares [15]. A comparable, later investigation in pony mares demonstrated that obese, insulin-resistant pony mares had higher serum TNF concentrations than those that were leaner and more insulin-sensitive [3]. These two investigations have provided supporting epidemiological evidence that obesity could be associated with increased inflammation in horses. However, as suggested by two recent studies, it is possible that a relationship between obesity and inflammation does not exist in horses. Suagee *et al*. reported that serum TNF concentrations were not altered in gelded horses while they were being fed to increase in adiposity over several months [16]. In that study, horses did not reach true obesity, and neither were they evaluated after being maintained in the overweight state. In a second study, serum TNF concentrations were found to be lower in obese, hyperinsulinemic horses than normal, lean controls [17]. Horses used for this investigation were aged similar to other studies; however, the investigation did not restrict gender or control diet. Therefore, we suggest that future investigations evaluating a potential influence of obesity on inflammation control for variables such as age, gender, and diet.

### 2.1. Role of Adipose Tissue

If obesity is correlated to increased concentrations of pro-inflammatory mediators, what is the source of these proteins? In humans, both adipose tissue and skeletal muscle produce pro-inflammatory cytokines. While the majority of research has focused on the contribution of adipose tissues to circulating cytokine concentrations, it is possible that production in skeletal muscle could also be very important to systemic glucose metabolism. 

Adipose tissue is present throughout the body in different storage locations, or depots, including intra-abdominal (mesenteric and omental) and subcutaneous, that require consideration. Intriguingly, recent research indicates that abdominal depots may be more “inflammatory” in humans than subcutaneous depots [18]. This potential for ‘depot-specific’ inflammation may be relevant to equine scientists because of the association between excess subcutaneous adipose deposited along the crest of the neck (regional adiposity) and elevated laminitis risk [2]. Furthermore, a recent investigation of adipose-depot-associated inflammation in overweight, but not obese, horses revealed that IL-1β and IL-6 mRNA are higher in subcutaneous adipose deposited along the crest of the neck than other depots including omental, retroperitoneal, mesenteric, and subcutaneous adipose from the tailhead [19]. These are important findings to keep in mind when reviewing equine data because subcutaneous adipose is the primary adipose depot investigated due to ease of sampling. 

Within adipose tissue, several cell types are capable of producing pro-inflammatory cytokines, including stromal-vascular cells (preadipocytes, fibroblasts, and non-differentiated mesenchymal cells), differentiated adipocytes, and infiltrated macrophages. When cell types are separated, TNF mRNA abundance is lower in differentiated adipocytes than the non-adipocyte fraction of adipose tissue from humans [20] and mice [21]. However, when differentiated mouse adipocytes were co-cultured with macrophages, TNF and IL-6 production by adipocytes was increased [22]. These results support the ability of differentiated adipocytes to produce pro-inflammatory cytokines, and further, that the up-regulation seen in obesity may require a signal from another cell type. In horses, the basal expression of cytokines from different cell types has not been thoroughly investigated; however, lipopolysaccharide (LPS) stimulation, which directly activates transcription of pro-inflammatory cytokines through the transcription factor nuclear factor-κB, has been used. In rump fat from two horses of unreported body condition, both adipocytes and pre-adipocytes had cytokine mRNA responses to LPS stimulation, although a greater percentage of pre-adipocytes stained for TNF than mature adipocytes [23]. These results could indicate that inflammatory responses of adipose tissue cell types are similar between horses and other species. In order to assimilate this new information with existing knowledge, further investigations and future reports need to include information on body condition and adipose depot. In conclusion, it appears that many cell types are capable of producing cytokines, and may do so constitutively at a low level, even in lean, healthy individuals. This raises an important question: what specific changes occur in the adipose tissue of obese animals that instigates low-grade, systemic, inflammation?

### 2.2. Role of Skeletal Muscle

As skeletal muscle is the major site of glucose disposal, myocyte production of cytokines with insulin desensitizing action could also contribute to the pathology of equine insulin resistance [24]. In humans, obesity increases pro-inflammatory proteins in skeletal muscle, similar to adipose tissue [25]. Skeletal muscle TNF and IL-6 mRNA are greater in diabetic humans, and TNF protein secretion from muscle is greater in cultured cells derived from diabetic humans [26,27]. It is possible that mechanisms for increased myocyte pro-inflammatory cytokine expression are similar to those mechanisms involved in adipose tissue. This includes evidence for mechanisms of increased macrophage infiltration, and hyperinsulinemia-induced cytokine production [28,29,30,31,32].

In horses, limited data on skeletal muscle inflammation are available. Following lipopolysaccharide (LPS) infusion, neither IL-1β nor TNF mRNA were increased, and IL-6 mRNA was undetectable 24 h following LPS administration in skeletal muscle [33]. As earlier time points have not been investigated, it is difficult to speculate about the role of skeletal muscle in the production of cytokines, either acting locally, or contributing to systemic concentrations. In opposition to the LPS study findings, a 6 h insulin infusion that induced acute, supraphysiological hyperinsulinemia also increased skeletal muscle IL-1β about 1.75 fold above baseline measurements [34]. Thus, neither the local production of cytokines by skeletal muscle nor the effects of cytokines on skeletal muscle have been characterized.

### 2.3. Mechanisms of Increased Pro-Inflammatory Cytokine Production

The majority of research into potential mechanisms stimulating increased circulating pro-inflammatory cytokine concentrations has been examined in adipose tissue. For this reason, the discussion below focuses on these mechanisms, including macrophage infiltration into adipose tissue, hyperinsulinemia, hypoxia of adipose tissue, and increased LPS. As previously mentioned, however, similar mechanisms may also occur in skeletal muscle.

#### 2.3.1. Macrophage Infiltration into Tissues

Adipose tissue macrophages are a potentially important source of cytokines [21,35], and also have the ability to increase cytokine production by other cell types. Although resident macrophages are present in all tissues as part of the innate immune system, an increased number of macrophages residing in the adipose tissue of obese humans is observed, and preliminary data suggest a similar relationship in horses [21,35,36,37]. Data derived from human studies indicate that adipocytes produce chemokines that signal monocyte and macrophage infiltration into adipose tissue. One of these chemokines, with limited data in horses, is monocyte chemoattractant protein-1 (MCP-1), which is responsible for attracting monocytes out of the bloodstream and into adipose tissue [38,39]. Serum concentrations of MCP-1 are significantly correlated to body mass index, waist circumference, and other inflammatory markers in humans, mRNA is increased in adipose tissue of obese mice, and an *intravenous *infusion of MCP-1 increases macrophage infiltration into mouse adipose tissue [39,40,41,42,43,44]. Thus, evidence indicates that MCP-1 is involved in recruiting macrophages to adipose tissue in obese humans and rodents; however, in non-obese, over-conditioned horses, MCP-1 mRNA is not different across adipose depots or altered by insulin sensitivity status [19]. This could indicate species-specific differences, or that an increase in MCP-1 is not seen until onset of obesity or following a prolonged period of obesity. Of further interest is the knowledge that MCP-1 expression is stimulated by TNF [45,46,47]; however, 24 h following the LPS infusion that increased plasma TNF, MCP-1 mRNA is not increased in subcutaneous adipose tissue of horses [33]. Adipose tissue MCP-1 expression might be increased earlier than 24 h, as serum TNF concentrations peak 1 h following LPS and return to baseline by 4 h. Alternatively, a localized inflammatory state in adipose tissue that is found with obesity was not induced by LPS infusion. The induction of obesity-associated adipose tissue inflammation in horses, akin to that observed in other species, might require more complex conditions than systemic inflammation affords.

In addition to assessing the potential for increased inflammation of adipose tissue macrophages, research has assessed inflammatory gene expression of circulating white blood cells. Intriguingly, Vick *et al*. reported a positive correlation between adiposity and IL-1β and TNF white blood cell mRNA; however, IL-6 mRNA was not related to adiposity [15]. This could indicate that IL-6 mRNA is not increased by obesity, or that circulating white blood cells in general, and more specifically, white blood cell IL-6 mRNA, are not indicators of adipose tissue inflammation. The capacity to assess adipose tissue inflammation through a blood sample would improve animal welfare by decreasing the need for adipose tissue biopsies; however, the validity of this approach requires further investigation.

#### 2.3.2. Hyperinsulinemia

Anecdotal observation suggests that high glycemic diets are commonly fed to horses and scientific evidence has demonstrated that these diets induce a postprandial increase in plasma insulin concentrations [48]. In humans, evidence exists for the ability of insulin to promote inflammation during acute, experimentally-induced hyperinsulinemia, independent of obesity, increasing inflammatory cytokine concentrations in subcutaneous adipose tissue interstitial fluid as well as circulating IL-6 concentrations [49,50]. These findings that suggest insulin somehow activates the production of cytokines are relevant to equine nutrition because of the demonstrated relationship between high glycemic diets and lower insulin sensitivity in obese horses [51]. Given this association, and the potential relationship between insulin, inflammation, and insulin sensitivity, Suagee et al. demonstrated that geldings consuming a high glycemic diet have increased serum TNF concentrations compared to geldings consuming a low glycemic diet [16]. As these horses were not obese, the higher TNF concentrations suggest high glycemic diets might promote inflammation independent of obesity. Using a more direct approach, hyperinsulinemia, achieved through a six-h insulin infusion, increased circulating TNF and IL-6 concentrations in non-obese mares [34]. However, even though white blood cell IL-6 mRNA was elevated, neck subcutaneous adipose tissue mRNA levels were not altered. Investigation of multiple adipose tissue depot responses to insulin is required. Other factors that could be involved in the insulin-inflammation axis include level of adiposity or degree of macrophage infiltration. Adipose tissue used to investigate insulin-induced inflammation was collected from obese humans, and this could account for some of the observed species differences. 

These *in vivo *data provide a good starting point for asking questions such as: which tissues are involved in insulin-induced inflammation in horses, and within those tissues, which cell types. As stated previously, acute hyperinsulinemia did not seem to create a pro-inflammatory state in neck subcutaneous adipose of horses; however, this effect is apparent in differentiated mouse and human adipocytes [52,53]. In these cells, the addition of insulin to culture media increased mRNA abundance and secretion of IL-6 and MCP-1. An exact mechanism describing the ability of insulin to promote inflammation is not yet described for all cytokines. However, the IL-6 promoter contains a response element for cAMP-response element binding (CREB) protein [54]. Insulin activates CREB via cGMP in human adipocytes, thereby increasing IL-6 transcript abundance [55]. Insulin also increased IL-6 protein synthesis and secretion through a MAPK dependent pathway [55]. The functionality of these pathways has not been investigated in horses.

#### 2.3.3. Hypoxia of Adipose Tissue

The possibility that adipose tissue hypoxia leads to increased inflammation involves the theory that expansion of adipose tissue mass may not be paralleled by increased vasculature. Adipose tissue hypoxia was first recognized in obese surgical patients, who had a greater risk of surgical site infections in association with low tissue oxygen tension [56]. It is possible that the greater metabolic activity and oxygen demand of infiltrating immune cells plays a role in adipocyte hypoxia, or that hypertrophied adipocytes have a larger diameter than the O_2_ diffusion distance, a fact that would increase the propensity that these cells exist under hypoxic conditions [57,58,59]. The physiological evidence for adipose tissue hypoxia includes impaired postprandial blood flow to adipose of non-obese insulin-resistant humans and hypoxia in adipose tissue of genetically obese (ob/ob) and diet-induced obese mice confirmed by use of interstitial O_2_ sensors [60,61,62]. *In vitro *hypoxia increases pro-inflammatory cytokine responses in human stromal-vascular cells as well as differentiated mouse adipocytes, and it is possible that a similar phenomenon could occur in equine adipose tissue [61,63].

The cellular response to hypoxia is highly conserved and serves to adapt cells to preservation of critical metabolic functions. These adaptations are primarily facilitated through activation and maintenance of the transcription factor, hypoxia-inducible factor-1 (HIF-1) [64]. Normal oxygen levels stabilize the enzymes that degrade the α-subunit of HIF-1, thus, only in low oxygen environments is HIF-1α capable of binding HIF-1β (constitutively expressed and stable) to form HIF-1 [65,66]. Stable HIF-1 binds to hypoxia response elements in the promoter region of several genes, and increases mRNA abundance and secretion of IL-1β, IL-6, TNF, and other pro-inflammatory mediators in adipose tissue [61,62,67,68,69]. Further, abundance of HIF-1α mRNA is increased in adipose tissue from obese humans [70]. Hypoxia also activates NFκB in adipocytes, which then promotes transcription of pro-inflammatory cytokines [61,71]. The activation of NFκB in response to hypoxia is possibly due, in part, to activation of the IκB kinase complex, similar to the mechanism of LPS stimulation, but could involve other mechanisms such as tyrosine phosphorylation of IκBα [72,73]. While it is likely that HIF-1 activation is a stronger response to hypoxia than NFκB, it is possible that NFκB may play a direct role in HIF-1 expression as HIF-1α contains an NFκB binding site in its promoter [73,74,75]. Thus, several feedback and feedforward signals appear to exist to promote and control cellular responses to hypoxia, but how these signals are moderated or influenced by obesity remains only partly explained. Hypoxia-induced inflammation is a viable theory that, to our knowledge, is uninvestigated in relation to equine obesity. 

#### 2.3.4. Increased Uptake of Gastrointestinal-Tract-Derived Lipopolysaccharide

Yet another potential theory explaining the relationship between obesity and inflammation involves a potential capacity for increased production and systemic absorption of hindgut microbial products, including the endotoxin, LPS. The gastrointestinal tract is designed to resist the crossing of bacterial products into the bloodstream. However, it is possible that high starch and sugar diets reduce the structural integrity of the intestinal epithelium, as a percentage of starch escapes the small intestine undigested and is fermented in the cecum and colon. In the cecum and colon, starch is fermented to lactic acid, which reduces cecal, colonic, and fecal pH values [76,77]. This situation is somewhat similar to that observed in ruminants with high starch diet-induced rumen acidosis. In these animals, epithelial tight junction integrity is compromised and increased plasma LPS concentrations are observed [78,79]. In horses, nasogastric administration of starch at values 7 to 10-fold greater than the recommended dietary maximums is a model used to induce laminitis [80,81,82,83,84]. Although this is an extreme scenario, the cecal pH values of horses subjected to carbohydrate overload were decreased from 7.0 to 4.7, and there was degeneration of the tight junctions between epithelial cells of the cecum [85]. In a similar experiment using elevated oligofructose administration, increased plasma LPS concentrations were reported [86]. It does not appear that LPS concentrations have been reported in horses consuming high starch and sugar concentrates at a level that would not directly induce laminitis. However, if consumption of these diets increases plasma LPS concentrations, then it might be possible that this is a mechanism relating these diets to inflammation and insulin resistance. 

The reason LPS production and uptake from the gastrointestinal tract is interesting is because LPS induces transient insulin resistance in horses 24 h after intravenous infusion [23,33,87], and also increases inflammatory protein production. In LPS-treated horses, white blood cell IL-1β, IL-6, and TNF mRNA and plasma TNF concentrations are increased within 1 h of infusion, while plasma SAA concentrations are increased at 3 h and remain elevated for at least 24 h [23,33,88]. Of further interest is the finding that IL-6 mRNA is elevated in adipose tissue at 24 h post infusion: the time point corresponding to insulin resistance. If increased uptake of LPS from the gastrointestinal tract occurs, then its potential influence on insulin resistance should be investigated. However, little equine research has focused on either the uptake or function of LPS outside of extreme models. For instance, while a bolus intravenous LPS infusion induces profound, transient insulin resistance, it is unknown how chronic, low-level plasma LPS concentrations might influence insulin resistance in obese horses.

## 3. Effects of Pro-Inflammatory Cytokines on Equine Metabolism

During sickness, increased pro-inflammatory cytokine production promotes physiological responses that facilitate the re-prioritization of energy expenditures to host defenses [89,90]. These responses include fever [91], fatigue, and loss of appetite [89,90]. As decreased energy intake occurs simultaneously to the increased energy expenditure of elevated body temperatures [92], fuel re-partitioning must occur. Glucose utilization by the immune system is also increased [93,94], requiring that non-immune cells reduce their use of available glucose. Non-immune tissues increase energy usage from non-glucose sources, and to support this, plasma-free fatty acids are increased during infection [95]. The altered tissue metabolism of sickness that increases energy availability to immune cells is due to the actions of pro-inflammatory cytokines. The response to illness is transient, and normal metabolism is restored once the animal recovers from infection. The chronic low grade systemic inflammation resulting from obesity may, however, cause more long-term alterations in metabolism, including both dyslipidemia and diabetes [14,96,97,98,99,100,101,102,103,104].

Pro-inflammatory cytokines impair glucose disposal by insulin sensitive tissues, thereby mediating insulin resistance. In adipose tissue and adipocytes, TNF, IL-6 and IL-1β have all been shown to have negative effects on insulin signaling. These include the *in vitro* effect of TNF to decrease mRNA and protein abundance of the insulin receptor, insulin receptor substrate (IRS)-1, and GLUT4, all of which result in reduced GLUT4 plasma membrane translocation [22,105,106,107]. Further, TNF increases serine phosphorylation of IRS-1, leading to proteosomal degradation and decreased ability of IRS-1 to promote insulin signaling [107,108]. In addition to the actions of TNF, IL-1β also reduces insulin-stimulated glucose uptake in adipocytes by decreasing GLUT4 protein abundance, IRS-1 protein abundance, insulin-stimulated phosphorylation of IRS-1, and insulin-induced membrane translocation [109,110]. In mice, IL-6 inhibits insulin-stimulated tyrosine phosphorylation of IRS-1 and IRS-2 [111]. In adipocytes, chronic culture with IL-6 decreases transcription of IRS-1 and GLUT4, while *in vivo*, plasma IL-6 concentration is inversely proportional to insulin-stimulated glucose transport into adipose tissue [112,113,114]. These data demonstrate the profoundly negative consequences of adipose tissue pro-inflammatory cytokine production on insulin signaling in adipose tissue. As inflammatory proteins also act in an endocrine manner in addition to their paracrine and autocrine effects [115], cytokines produced by and released from adipose tissue could act systemically on tissues such as skeletal muscle.

Skeletal muscle insulin responsiveness is the largest component of peripheral insulin sensitivity, and any impairment of glucose utilization in skeletal muscle will have significant impacts on whole body glucose metabolism [24]. The pro-inflammatory cytokines, TNF, IL-6 and IL-1β have the ability to reduce insulin-stimulated glucose disposal into skeletal muscle, although less attention has been paid to this than to the metabolic effects of cytokines on adipose tissue. *In vivo*, TNF reduces glucose uptake into human skeletal muscle [116]. Use of rat-derived muscle cell lines showed this reduced glucose uptake to be due to inhibition of insulin-stimulated activation of the insulin signaling cascade, partly due to altered phosphorylation of several signaling intermediates [117,118,119]. In a mouse muscle cell line, TNF, but not IL-1β, reduced both glucose uptake and Protein Kinase B (PKB) levels [120]. Similar to TNF, IL-6 treatment also reduces skeletal muscle glucose utilization *in vivo *in mice, and treating a rat muscle cell line with IL-6 decreased insulin stimulated Akt phosphorylation [121,122].

In horses, the role of cytokines to directly and specifically alter metabolism in either adipose or muscle tissue have not yet been investigated. This information could be determined by culturing adipose tissue and muscle with equine cytokines, or through intravenous infusion of equine cytokines. This information could lend support for further research into strategies to prevent and treat insulin resistance by enabling scientists to focus on specific inflammatory markers. 

## 4. Conclusions

Insulin resistance in horses is associated with increased risk of laminitis. Thus, research efforts to identify causative factors of insulin resistance are important. In other species, increased production of pro-inflammatory cytokines during obesity is known to directly influence decreased insulin sensitivity, thus evidence of this relationship in horses has been evaluated. Although a correlation between plasma concentrations of TNF and obesity are described in horses, the tissue primarily responsible for production of this inflammatory protein has not been identified. Potential tissue sites of increased production of cytokines include several adipose tissue depots, and within adipose tissue there may be cell-specific roles in inflammation. In horses, subcutaneous adipose tissue from the neck has greater basal inflammation than other adipose depots, and stromal-vascular cells have greater response to inflammatory stimulation than mature adipocytes. Potential mechanisms responsible for stimulating cytokine production in adipose tissue include macrophage infiltration, hyperinsulinemia, hypoxia, and LPS. Of these, hyperinsulinemia and LPS stimulation have received the greatest attention. Future work should clearly determine whether inflammation is increased in adipose tissue of obese horses, and further, the role of diet to influence inflammation. This could involve direct investigation of insulin’s ability to stimulate inflammatory protein production from different adipose tissue depots, or the ability of diet to influence LPS concentrations. Increasing knowledge of these mechanisms will hopefully enable strategies for preventing and treating insulin resistance. Additionally, with more information we can focus research efforts on a specific adipose depot, which could potentially improve animal welfare, as fewer tissue samples will be required, or provide an incentive to produce a clonal adipocyte cell line.

Improving our understanding of the potential role of inflammation in the relationship between obesity and insulin resistance will hopefully allow for the development of strategies for preventing laminitis in obese horses. These strategies could include diet and pharmacologic agents, in addition to defining a specific marker of increased risk. Specifically, the individual roles of pro-inflammatory cytokines to modulate insulin sensitivity in skeletal muscle and adipose tissue should be identified to determine whether inflammation is indeed active in metabolic diseases similar to that observed in humans. If inflammation is influencing insulin sensitivity in horses, this will provide an incentive for increased resources to be allocated to this research, but if horses are unlike humans in response to cytokines, then research into other mechanisms relating obesity to insulin resistance should be explored.
